# Assessment of the potential for carbon sink enhancement in the overlapping ecological project areas of China

**DOI:** 10.3389/fpls.2024.1482077

**Published:** 2024-11-26

**Authors:** Xiaojuan Xu, Fusheng Jiao, Dayi Lin, Jie Qiu, Changxin Zou, Kun Zhang

**Affiliations:** ^1^ Nanjing Institute of Environmental Sciences, Ministry of Ecology and Environmental of the People's Republic of China, Nanjing, China; ^2^ School of Geography, Nanjing Normal University, Nanjing, China

**Keywords:** carbon sinks, prediction, climate change, stability, ecological projects

## Abstract

Ecological engineering can significantly improve ecosystem carbon sequestration. However, few studies have projected the carbon sink trends in regions where ecological engineering projects overlap and have not considered the different climate change conditions and land use scenarios. Using the ensemble empirical mode decomposition method and machine learning algorithms (enhanced boosted regression trees), the aims of this study to elucidate the stability of carbon sinks and their driving mechanisms in areas where ecological projects overlap and to predict the potential enhancement in carbon sinks under varying climate and human activity scenarios. The findings revealed that: (1) The carbon sinks clearly and steadily increased in regions where five ecological projects were implemented from 1982 to 2019. In contrast, the carbon sinks did not significantly increase in regions with two or three ecological projects. (2) As the number of ecological projects increased, the impact of human activities on the carbon sinks gradually decreased. In eastern China, rapid economic development and significant interference from human activities hindered the growth of carbon sinks. In contrast, in western China, the warming and humidification trend of the climate, large-scale afforestation, and other ecological projects have significantly improved carbon sinks. (3) The regions with five overlapping ecological projects exhibited the greatest enhancement and stability of carbon sinks under different scenarios. Compared with the SSP585 scenario, under the SSP126 scenario, the carbon sinks increased, and their stability was greater. Achieving carbon neutrality requires major ecological projects to account for the limitations imposed by climatic conditions. Instead of isolated projects or the implementation of single restoration measures, a comprehensive approach that uses the synergistic effects of combined ecological strategies is recommended.

## 1 Introduction

Ecological engineering is critical for enhancing terrestrial carbon sinks, which are essential for mitigating climate change through the absorption of carbon dioxide (CO_2_) and its sequestration in terrestrial ecosystems (Zhang et al., 2022; Niu et al., 2023; Li et al., 2024; Xia et al., 2024). Since the 1980s, China has initiated several significant ecological projects, including the Three North Shelterbelt Forestation Project, the Grain to Green Project, the Natural Forest Resource Protection Project, and the Desertification Control in the Karst Region of Southwest China Project (Yang et al., 2022; Tong et al., 2023). Currently, 17.42% of China is influenced by a single ecological engineering project, while 78.87% is affected by multiple ecological engineering projects (Shao et al., 2023). However, previous studies have mainly focused on the trends and driving mechanisms of carbon sinks under individual ecological engineering projects, and little attention has been paid to the combined effects of multiple overlapping projects. Furthermore, the prospects for carbon sequestration and the enhancement of carbon sinks within overlaying ecological engineering project areas remain ambiguous. In addition, it remains unclear whether overlapping ecological projects can contribute more to the increase in carbon sinks than single ecological projects, and whether the carbon peaking and carbon neutrality targets can be realized in the near future (Zhao et al., 2021). Thus, it is necessary to assess the stability and driving mechanisms of carbon sinks, and to predict their potential enhancement under different scenarios in overlapping ecological engineering project areas (Yue et al., 2020).

Ecosystem stability is crucial for regulating the terrestrial carbon cycle and atmospheric CO_2_ levels (Messori et al., 2019; Arani et al., 2021). Understanding the stability of terrestrial ecosystems can improve the accuracy of predictions of ecosystem changes and provide information for developing climate change mitigation policies (Seddon et al., 2016; Pennekamp et al., 2018; Wang et al., 2022b). The main function of carbon sinks is to absorb CO_2_, thereby reducing greenhouse gas concentrations in the atmosphere. Stabilized increasing carbon sinks can effectively store CO_2_ over a long period, mitigating the increase in global temperatures (Piao et al., 2020; Wang et al., 2022a). An unstable trend of carbon sinks may lead to the release of CO_2_, further exacerbating climate change and leading to ecosystem imbalance (Liu et al., 2023a; Wang et al., 2024). However, due to the increasing frequency of extreme weather events driven by climate change, interannual fluctuations in carbon sinks are increasing, indicating a decline in ecosystem stability (Holden et al., 2014; Hu et al., 2021).

Understanding the driving mechanisms of the stability of carbon sinks is crucial for the effective implementation of carbon neutrality policies (Liu et al., 2023b; Zhang et al., 2023b). Previous research has shown that temperature, precipitation, and radiation can directly influence vegetation photosynthesis and the carbon sequestration capacity (Wang et al., 2022b; Jin et al., 2023). A low soil moisture content and high atmospheric vapor pressure deficit (VPD) are the main driving factors of water stress and contribute to the reduction of carbon sinks (Li et al., 2022). In recent years, engineered water shortages have occurred frequently, exacerbating drought stress and thus limiting the stability of carbon sinks. Studies have shown that economic development and rural population reduction have promoted vegetation restoration and increased carbon sequestration (Chang et al., 2023). Additionally, China has implemented to several ecological engineering projects to enhance carbon sequestration, such as rocky desertification control, the Grain for Green Project, and the Natural Forest Protection Project (Tong et al., 2023). However, previous studies have rarely considered the combined impacts of climate change, human disturbances, and ecological restoration projects (Zhu and Lo, 2022; Chang et al., 2023). These factors, whether individually or interactively, affect the stability of carbon sink. Studies have indeed explored the relative importance of drivers of carbon sink trends (Wang et al., 2023; Jiao et al., 2024). However, the driving mechanisms for the stability of carbon sinks, particularly, in overlapping ecological engineering areas, remain unclear (Thackeray et al., 2016; Li et al., 2022).

Predicting the stability of carbon sinks is crucial for enhancing ecosystem carbon sequestration capacity and identifying areas at risk of carbon sequestration degradation (Yazdandoost et al., 2021; Xu et al., 2023a). Previous studies have shown that if the atmospheric CO_2_ concentration peaks at around 2060 and remains stable (carbon neutrality scenario), the global terrestrial carbon sink will gradually decrease starting around 2060, with an estimated reduction of 50% by 2100 (Zhang and Hanaoka, 2021; Zhang et al., 2023a). The rate of China’s terrestrial carbon sink is increasing, and it is expected to approach zero by 2100 (carbon neutrality scenario). Current studies have primarily predicted fluctuations in the carbon sequestration rates, but often few long-term predictions of carbon sink trends and stability have been conducted (Cai et al., 2022; Xu et al., 2022a). The prediction of carbon sink trends and stability can reveal whether China’s carbon sinks can maintain carbon neutrality, and lays the foundation for the development of theoretical frameworks to support carbon emission mitigation strategies and climate change adaptation plans (Zheng et al., 2020; Yazdandoost et al., 2021). These projections form the foundation for developing theoretical frameworks that support carbon emission mitigation strategies and formulate climate change adaptation plans (Zhang and Hanaoka, 2021). China’s key ecological engineering zones have extensive distributions, covering various geographic landscapes and climate systems (Shao et al., 2023). In ecologically fragile and economically underdeveloped areas, there is clear opposition between ecological preservation and resource utilization (Wang et al., 2024). This dichotomy introduces a layer of uncertainty into the prediction of carbon sink stability. Furthermore, there remains a pressing need for comprehensive assessments that address the risks associated with carbon sink degradation. This opposition introduces a layer of uncertainty in the prediction of carbon sink stability. Therefore, it is necessary to predict the stability of China’s carbon sinks for 2060 and 2100 under different climate and land use change (LUC) scenarios.

Based on the ensemble empirical mode decomposition (EEMD) method and machine algorithm (enhanced boosted regression tree (BRT)), in this study, we explored to explore the nonlinear trends and stability of carbon sinks from 1982 to 2019 in overlapped ecological engineering project areas in China. In addition, the trend and stability of carbon sinks for 2060 and 2100 were predicted under different climate change and human activity scenarios. More specifically, we (i) assessed the stability of carbon sinks in China from 1982 to 2019; (ii) investigated the driving mechanisms for the stability of carbon sinks; and (iii) predicted the future trends and stability under different shared socioeconomic pathway scenarios (SSP126 and SSP585).

## 2 Material and methods

### 2.1 Study area

**Figure 1:**
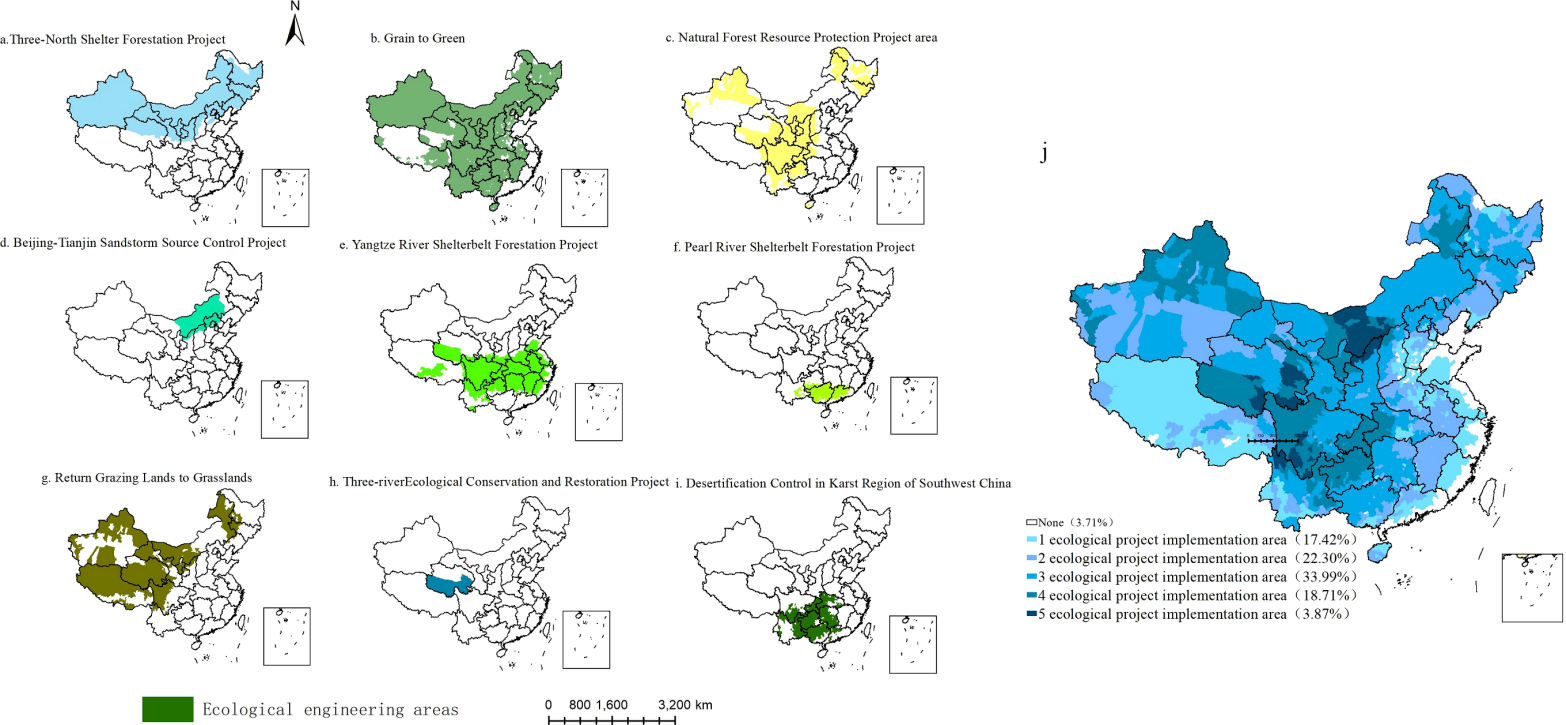
The spatial distribution of the single and overlapping ecological engineering projects. (**A**. the Three North Shelterbelt Forestation Project, **B**. the Grain to Green Project, **C**. the Natural Forest Resource Protection Project, **D**. the Beijing-Tianjin Sandstorms Source Control Project, **E**. the Yangtze River Shelterbelt Forestation Project, **F**. the Pearl River Shelterbelt Forestation Project, **G**. Return Grazing to Grassland Project, **H**. the Three-river Ecological Conversation and Restoration Project, **I**. the Desertification Control in Karst region of Southwest China).

China has launched nine key national engineering projects (**Figure 1**). These projects cover an extensive ecological engineering zone, with a total area of approximately 9.3 million km^2^. According to the spatial distributions of the implementation scope of the nine ecological engineering projects (**Figures 1A–I**), 96.29% of the regions are ecological engineering implementation areas. Only 3.71% of the regions remain unaffected by any ecological project, and project are primarily concentrated along the southern coast of China. The areas of overlapping ecological projects account for 78.87% of the national land area. Notably, three overlapping projects, namely, the Grain to Green Project, the Yangtze River Shelterbelt Project, and the Desertification Control in the Karst Region of Southwest China Project, cover the largest area encompassing 33.99% of China’s land and are mainly located in central China. The largest area of overlapping projects includes five ecological projects, covering 3.87% of China’s land area, primarily in the Three-River region of Qinghai, central and western Inner Mongolia, northern Shaanxi, southwestern Sichuan, and northern Yunnan.

### 2.2 Data source

Net Ecosystem Productivity (NEP) is a key indicator in discerning whether an ecosystem functions as a carbon sink or source. When NEP surpasses zero, it denotes that the fixed carbon outweighs the carbon consumed by heterotrophic respiration, thereby designating the ecosystem as a carbon sink. Conversely, when NEP falls below zero, it signifies that the fixed carbon is less than the carbon consumed by heterotrophic respiration, categorizing the ecosystem as a carbon source (Piao et al., 2022a). Thus, NEP greater than zero is adopted as the criterion for identifying a carbon sink. The dataset was sourced from the Resource and Environment Data Platform with a spatial resolution of 0.072°from 1981 to 2019 (http://www.nesdc.org.cn/). The NEP data were obtained from the mechanistic ecological boreal ecosystem productivity simulator (BEPS) driven by remote sensing vegetation parameter (leaf area index, aggregation index, and surface coverage) data, meteorological data, soil texture data, and atmospheric CO_2_ concentration data (Chen et al., 2019).

The climate change data, including air temperature (TEM), precipitation (PRE), soil moisture (SOIL), VPD, and downward shortwave radiation (RAD) data, were provided by the Climatology Lab (Abatzoglou et al., 2018). This climatic product has multiple data sources, including WorldClim, the Climate Research Unit, and the Japanese 55-year Reanalysis dataset. To enhance the spatial resolution, the multivariate adaptive constructed analogs method was used to downscale the monthly time series. This method has been proven to be superior to direct daily interpolated bias correction, particularly in regions with complex topography (Jiao et al., 2024). The climatic datasets provide monthly climate and climatic water balance information for terrestrial surfaces worldwide, covering the period from 1958 to 2020. The data have a spatial resolution of 1/24 of a degree, approximately equivalent to 4 km.

The human activities considered in this study included the LUC, population density (POP), gross domestic product (GDP), and anthropogenic emissions of CO_2_ and N_2_O. The land use data, obtained from the Resource and Environment Data Platform (http://www.resdc.cn/), were for 1980, 1990, 1995, 2000, 2005, 2010, 2015, and 2020 and had a spatial resolution of 30 m (Liu et al., 2018). The land use types were divided into five types: farmland, forest, grassland, urban and rural land, and other land. The LUC data were derived using the transfer matrix method. The GDP and population density data, obtained from the Resource and Environment Data Cloud Platform (http://www.resdc.cn/), were for 1995, 2000, 2005, 2010, 2015, and 2020 and had a spatial resolution of 30m. The anthropogenic CO_2_ and N_2_O emission data were obtained from the Global Atmospheric Research Emissions Database, covered the period from 1981 to 2019, and had a spatial resolution of 0.1°.

The future climate variables under different scenarios (SSP126 and SSP585) from 2020 to 2100 were provided by Phase 6 of the Coupled Model Intercomparison Project (CMIP6) (https://esgf-node.llnl.gov/search/cmip6/) (Zhang et al., 2023a). The future population and economy data under different scenarios (SSP126 and SSP585) were provided by Chen et al. (2020) with a resolution of 0.1° grid and encompasses a timespan from 2020 to 2100 (Chen et al., 2020) (https://www.nature.com/articles/s41597-020-0421-y).

All of the data were resampled to 0.072°, consistent with the carbon sink data.

### 2.3 Trend of carbon sinks

**Figure 2:**
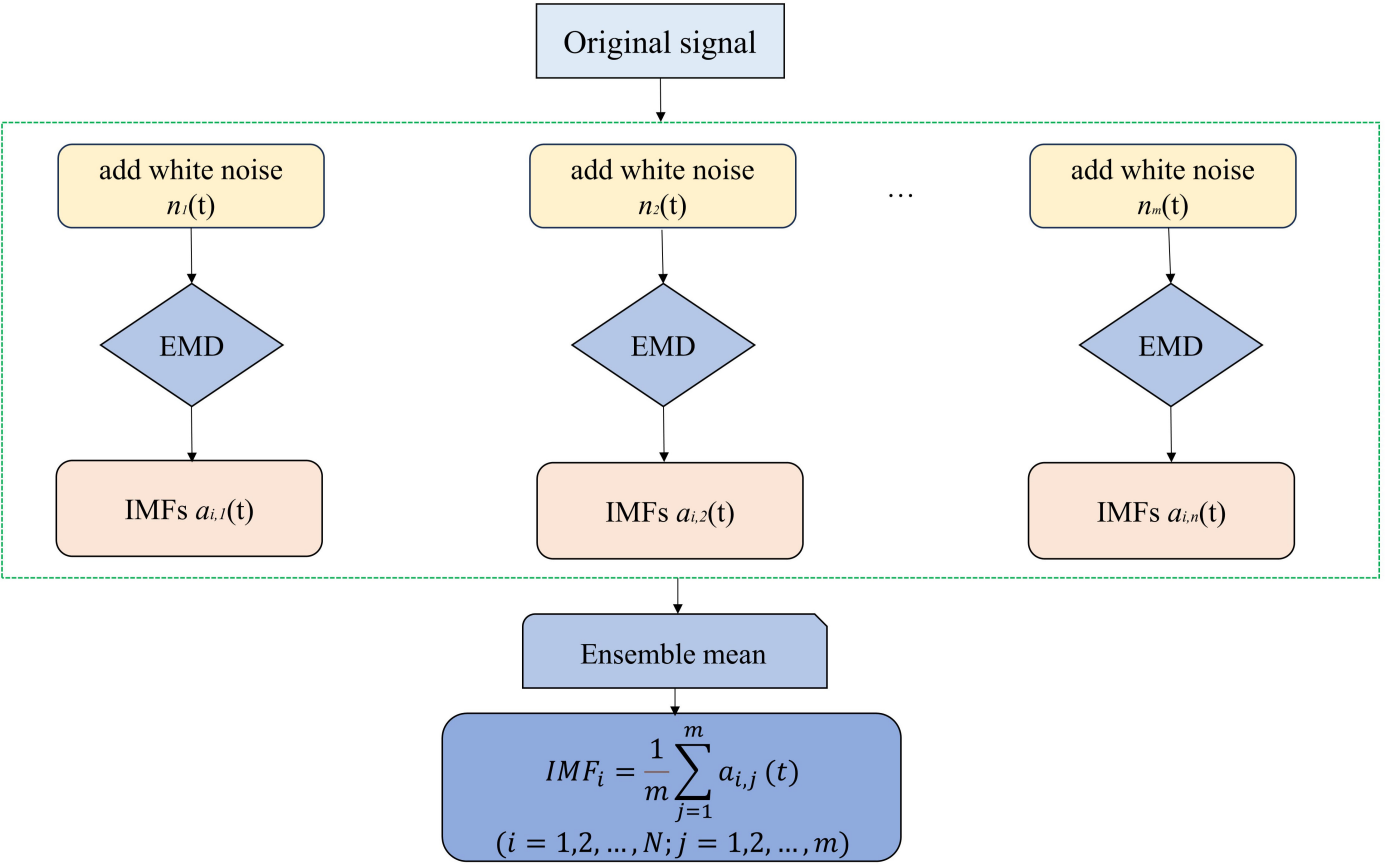
EEMD decomposition of long-time series process.

**Figure 3:**
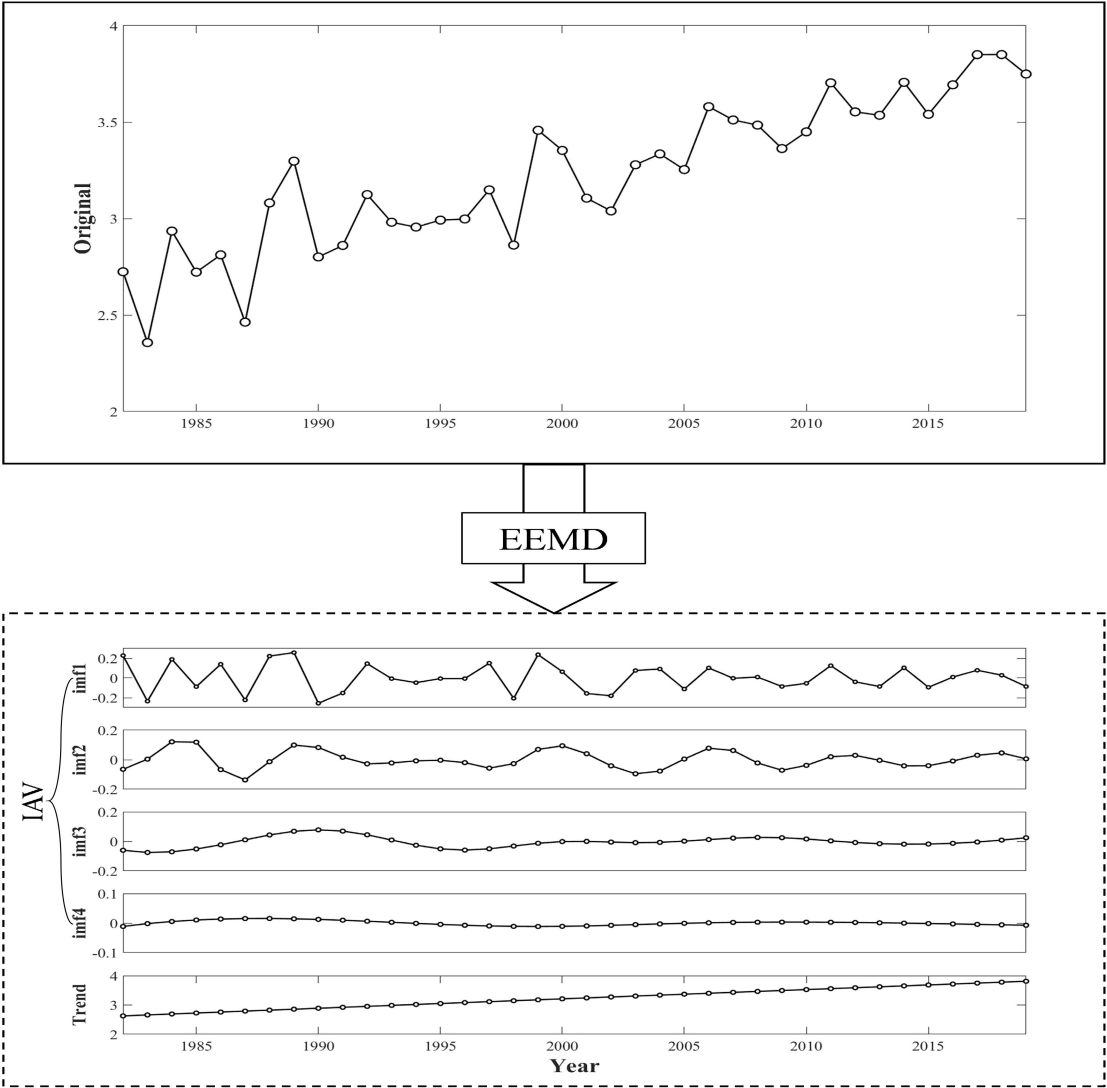
EEMD decomposition of inter-annual fluctuations (IVA) and trend terms (Trend) for carbon sinks.

The EEMD method is an advanced data analysis technique that decomposes complex signals into a finite number of intrinsic mode functions (IMFs) and residual components (**Figures 2**, **3**) (Wu et al., 2007). It improves the Empirical Mode Decomposition (EMD) method by addressing limitations such as mode mixing and boundary effects (Pan et al., 2018). EEMD efficiently handles non-smooth and nonlinear signals, making it ideal for analyzing time-series data across various domains (Ji et al., 2014). Its adaptability and robustness have led to its widespread application in signal processing, trend extraction, and feature analysis (Xu et al., 2022b). Furthermore, by identifying and quantifying intrinsic periodicities and trends within ecosystems, EEMD facilitates the assessment of the ecosystem’s responsiveness to external perturbations, thereby elucidating the ecosystem’s stability.

**Figure 4:**
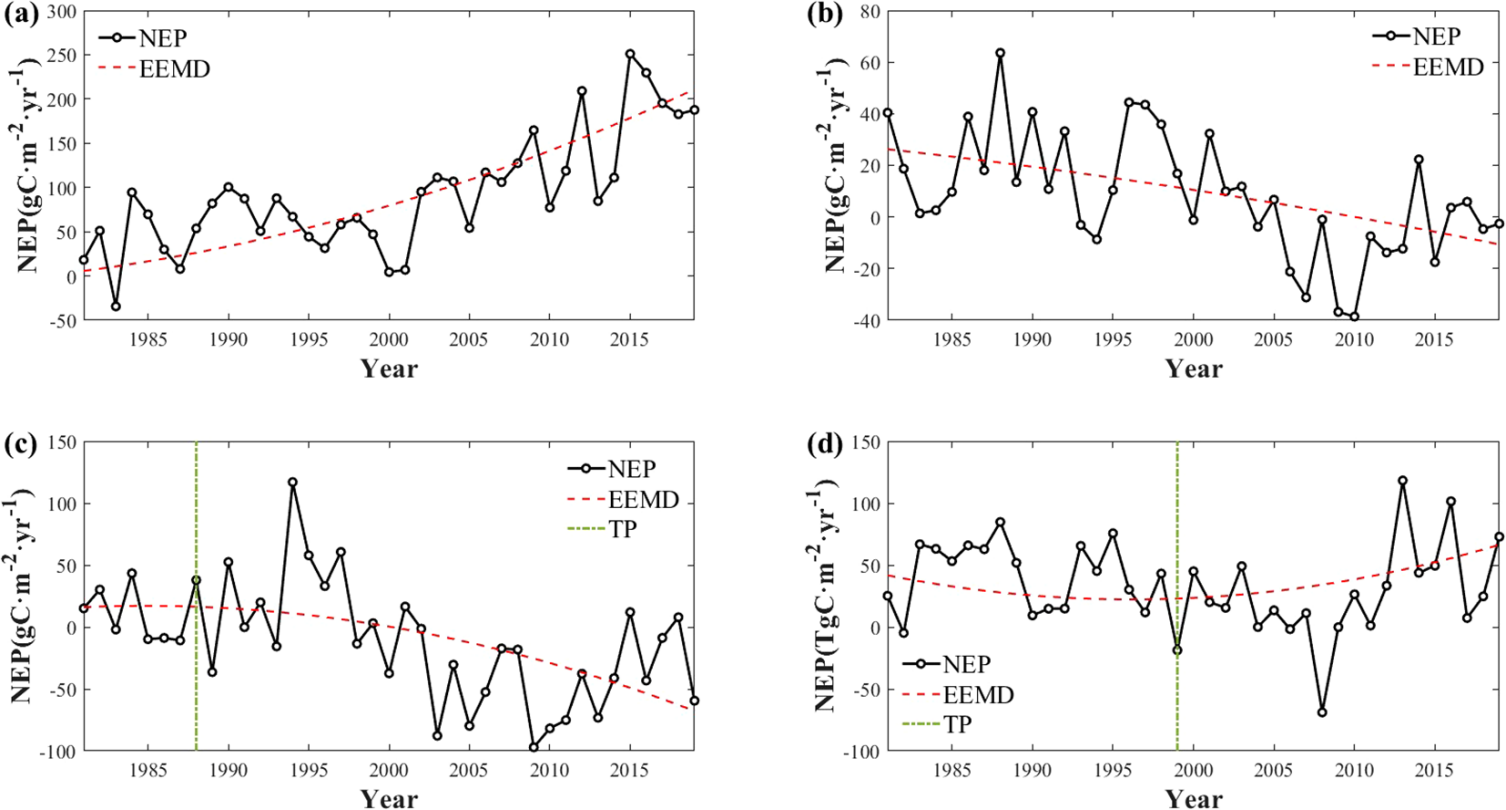
Four significant non-linear trends in EEMD-decomposed carbon sinks [**(A)** monotonically increasing; **(B)** monotonically decreasing; **(C)** negative shifts; **(D)** positive shifts, with the TPs are turning points].

The EEMD method involves adding multiple realizations of white noise, known as ensemble members, to the original signal to suppress noise and improve the decomposition accuracy. Each ensemble member undergoes individual processing via EMD, resulting in multiple IMFs. The final IMFs and residual components are derived by averaging the IMFs of all of the ensemble members (Ji et al., 2014). In this study, EEMD was used to extract the residual components of carbon sinks and to calculate the rates of change of these components. This analysis identified one significant trend and four types of trends of carbon sinks: increasing, decreasing, decreasing to increasing (positive reversals), and increasing to decreasing (negative reversals) (**Figure 4**).

### 2.4 Stability of carbon sinks

The stability of NEP was assessed by calculating the coefficient of variation (CV) of the detrended data (Zhang et al., 2019; Piao et al., 2020). The EEMD method was used to remove the trends from the NEP data, ensuring that the analysis focused on the inherent variability of the NEP data rather than the long-term trends (Lin et al., 2023).


$$NEP_{\det rend,year}\;=\;NEP_{year}\;-NEP_{trend,year}\;+{\text{ NEP}}_{mean\;}$$

$$NEP_{trend,year}\;=\;a\times year+b$$

$\;NEP_{year}$ is the raw annual NEP data. $NEP_{trend,year}$ is the NEP trend for each year, and the coefficients are calculated using a linear method. The mean $NEP_{mean\;}$ is averaged from $NEP_{year}$ . The detrended variable for each year is denoted as $NEP_{\det rend,year}$ . *NEP_CV_* is calculated using these detrended data:


$$NEP_{CV}=\frac{\sqrt{\frac{\sum {\left(NEP_{\det rend,year}-NEP_{mean\;}\right)}^{2}}{N}}}{NEP_{mean\;}}$$

The larger $NEP_{CV}$ is, the more volatile and less stable the NEP trend is. In this study, *NEP_CV_* was classified into five levels: stable, relatively stable, moderately stable, relatively unstable, and unstable. The natural interval method, which relies on inherent data groupings, was used to conduct this classification. This method clusters similar values together while maximizing the differences between classes, ensuring that the elements are categorized based on significant discrepancies in the data values.

### 2.5 Enhanced boosted regression tree

The enhanced boosted regression tree (BRT) method has a robust framework for analyzing complex ecological data and making reliable predictions (Elith et al., 2008). This method’s flexibility in handling nonlinear relationships and its ability to incorporate interactions among predictors make it an indispensable tool in ecological and environmental research (Czarniecka-Wiera et al., 2020). It constructs multiple regression trees through iterative random sampling and self-learning, evaluates the influence of the independent variables on the dependent variable, and uses the remaining data for cross-validation. This approach is not constrained by the temporal sequence of data, thereby increasing its robustness. The BRT method is proficient in identifying key variables, nonlinear relationships, and the interactions between and relative importance of factors influencing the stability of carbon sinks. In this study, the relative importance of human activities (anthropogenic emissions of CO_2_ and N_2_O, LUCs, economic development, and population density) and climate change (temperature, precipitation, soil moisture, atmospheric VPD, and solar radiation) on the stability of carbon sinks was extracted using the BRT method.

In this study, a subsample of 75% was selected from the carbon sinks as a training sample, and the rest were used as validation samples. A 10-fold cross-validation method was executed for the BRT model. Meanwhile, 10 iterations were run on the BRT model to reduce model uncertainty and all results were averaged. To measure the model accuracy, the AUC was used to validate the fitting accuracy between the carbon sink stability and the driver. Model accuracy can be judged as excellent if AUC ≥ 0.9, good if 0.8< AUC< 0.9, accurate if 0.7< AUC< 0.8, and poor if 0.6< AUC< 0.7. As shown in **Supplementary Table S1**, the average AUC value of 0.88 > 0.8 indicates good fitting accuracy for carbon sink stability.

In addition, the BRT model enabled the simulation and prediction of the trend and stability of carbon sinks across various scenarios, offering insights into their sustainability. The iterative boosting approach improves the predictive performance by combining multiple weak models to form a strong predictive model (Elith et al., 2008). Drawing upon data for two shared socioeconomic pathways (SSP126 and SSP585) provided by the CMIP6, in this study, we used the BRT model to forecast the long-term trends and stability of carbon sinks within the ecological engineering zones for 2060 and 2100 under different climate and human activity scenarios. We used this model to classify the significant increases and positive shifts in the trends of carbon sinks as the carbon sink increases and decreases and the negative shifts as the carbon sink decreases. Furthermore, it was used to identify future ecological restoration areas and key protection zones, as well as to investigate nature-based carbon sequestration management models.

## 3 Results

### 3.1 Trend of carbon sinks in overlapping ecological project areas

**Figure 5:**
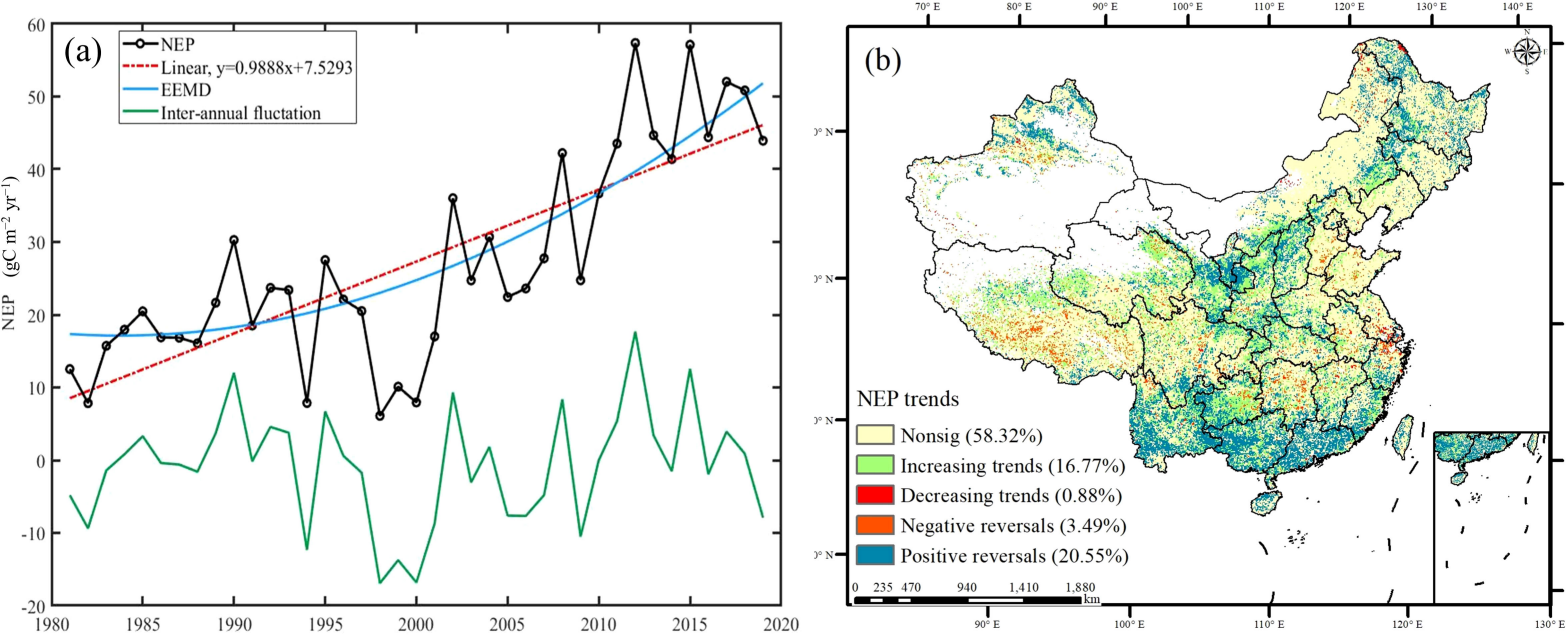
The nonlinear trends of carbon sinks in China. (**A**: the linear nonlinear trends of carbon sinks, **B**: the spatial distribution of nonlinear trends in carbon sinks).

China’s carbon sinks exhibited a steadily increasing trend, with a growth rate of 0.9888 gC m^−2^ yr^−1^ (**Figure 5A**). Moreover, using the EEMD method, it was found that the carbon sinks exhibited a nonlinear increasing trend. Before 1995, the carbon sink fluctuations increased. After 1995, the carbon sinks experienced rapid growth. This suggests an accelerating trend of carbon sink enhancement. The interannual fluctuations in the carbon sinks ranged from −16.81 to 17.68 gC m^−2^ year^−1^. Smaller fluctuations occurred before 2000, and larger fluctuations occurred after 2000. Consequently, it is imperative to elucidate the changes in the stability of the carbon sinks.

On a spatial scale, the significant trends in the carbon sinks covered 41.68% of China (**Figure 5B**). The increasing trends and positive reversals of the carbon sinks constituted 16.77% and 20.55%, respectively, while the decreasing trends and negative reversals accounted for only 0.88% and 3.49%, respectively (**Figure 5**). The areas exhibiting increasing trends of carbon sinks primarily occurred in the northern part of the Tibetan Plateau and the southwestern region. The positive reversals predominantly occurred in the southwest region, southeast coast, Loess Plateau, and northeastern region. The increasing trends and positive trends of carbon sinks were mainly concentrated in areas where the Grain to Green, Returning Pasture to Grassland Project, and Rocky Desertification Control projects overlapped. Conversely, the decreasing trends and negative reversals primarily occurred in the Tibetan Plateau, Yangtze River Basin, and northern plain areas (**Figure 5B**).

**Figure 6:**
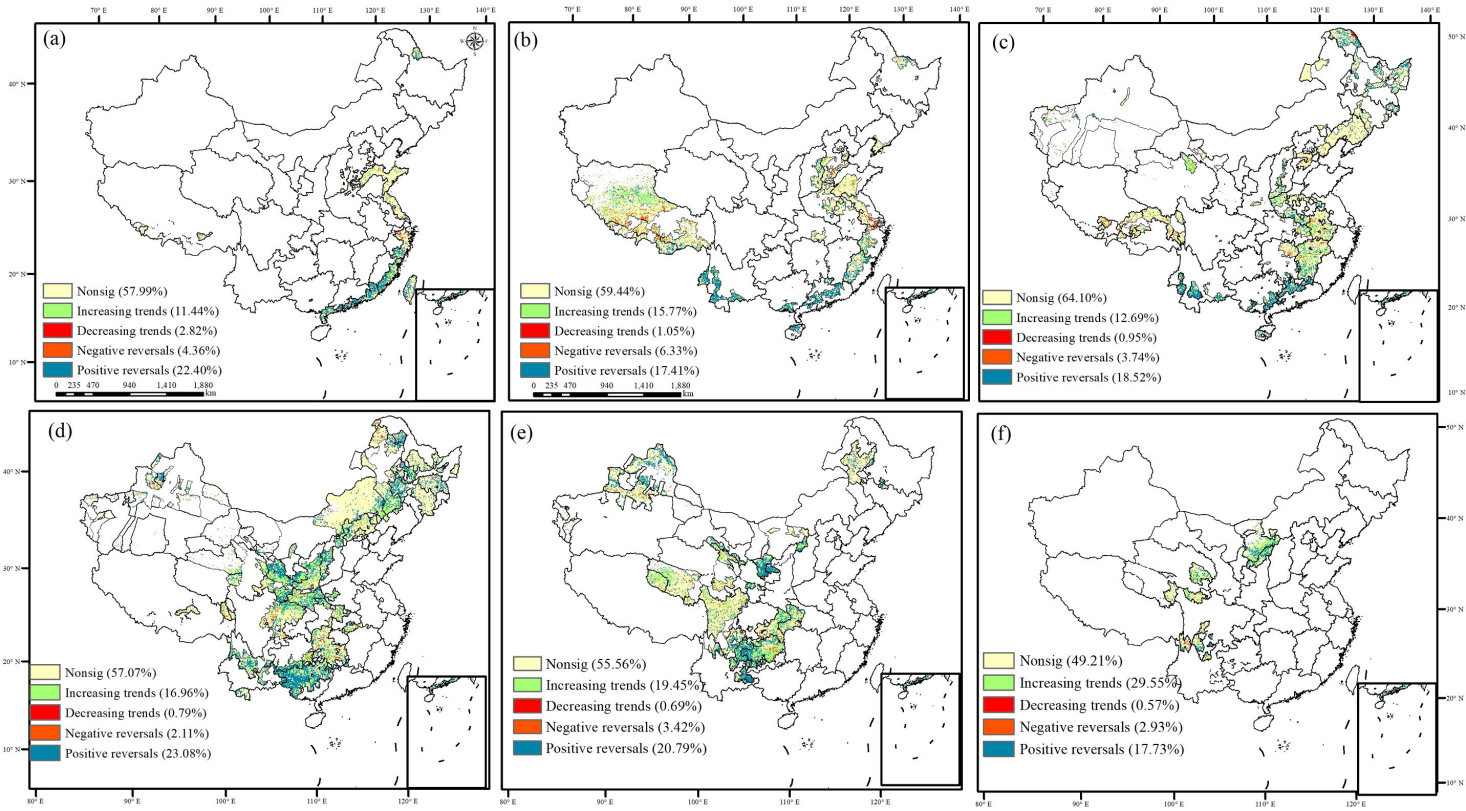
The trend of NEP from 1981 to 2019 in ecological project areas [**(A)** 0 ecological project areas; **(B)** 1 ecological project area; **(C)** 2 ecological project areas; **(D)** 3 ecological project areas; **(E)** 4 ecological project areas; **(F)** 5 ecological project areas].

In the ecological engineering areas, the proportion of increasing trends of carbon sinks increased as the number of overlapping ecological projects increased (**Figure 6**). The carbon sinks increased in 29.55% of the regions where five ecological engineering projects overlapped, such as the Grain to Green, Natural Forest Resource Protection Projects, Return Grazing Lands to Grasslands, and Desertification Control in the Karst Region of Southwest China projects. Conversely, as the number of ecological engineering projects increased, the proportion of decreasing trends and negative reversals of the carbon sinks decreased. Notably, the proportion of positive reversals of the carbon sinks did not consistently increase as the number of overlapping ecological engineering projects increased. It peaked in areas where three ecological engineering initiatives were implemented. These areas, primarily located in Southwest China, the Yellow River Basin, and the Northeast China Plain, overlapped with projects such as the Grain to Green, Natural Forest Resource Protection Project, Three-North Shelter Forestation Projects, and Desertification Control in the Karst Region of Southwest China Project areas.

### 3.2 Stability of carbon sinks in overlapping ecological project areas

**Figure 7:**
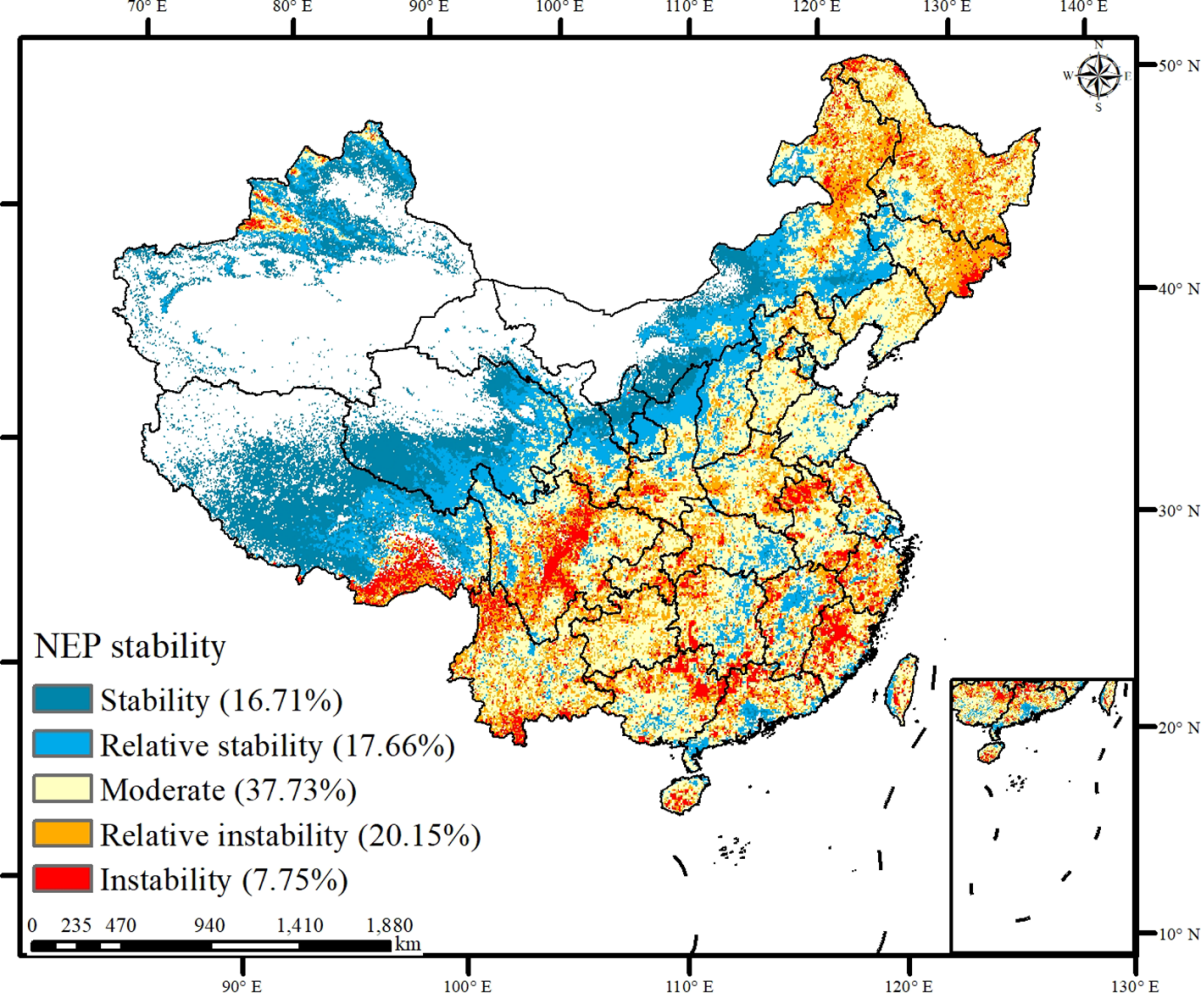
The stability of carbon sinks in China.

The spatial distribution of the stability of carbon sinks across China exhibited notable variations (**Figure 7**). Regions such as the South Karst area, Southeast Hill area, North China Plain, and Northeast China Plain exhibited larger interannual fluctuations in the carbon sinks, indicating instability. Moreover, these areas exhibited increasing trends or positive reversals alongside significant carbon sinks. The carbon sinks were stable in northern Xinjiang and the southern part of the Tibetan Plateau. However, despite this stability, these regions experienced insignificant changes or negative reversals of the carbon sinks. Thus, the stability of the carbon sinks did not consistently correspond with their increasing trends.

**Figure 8:**
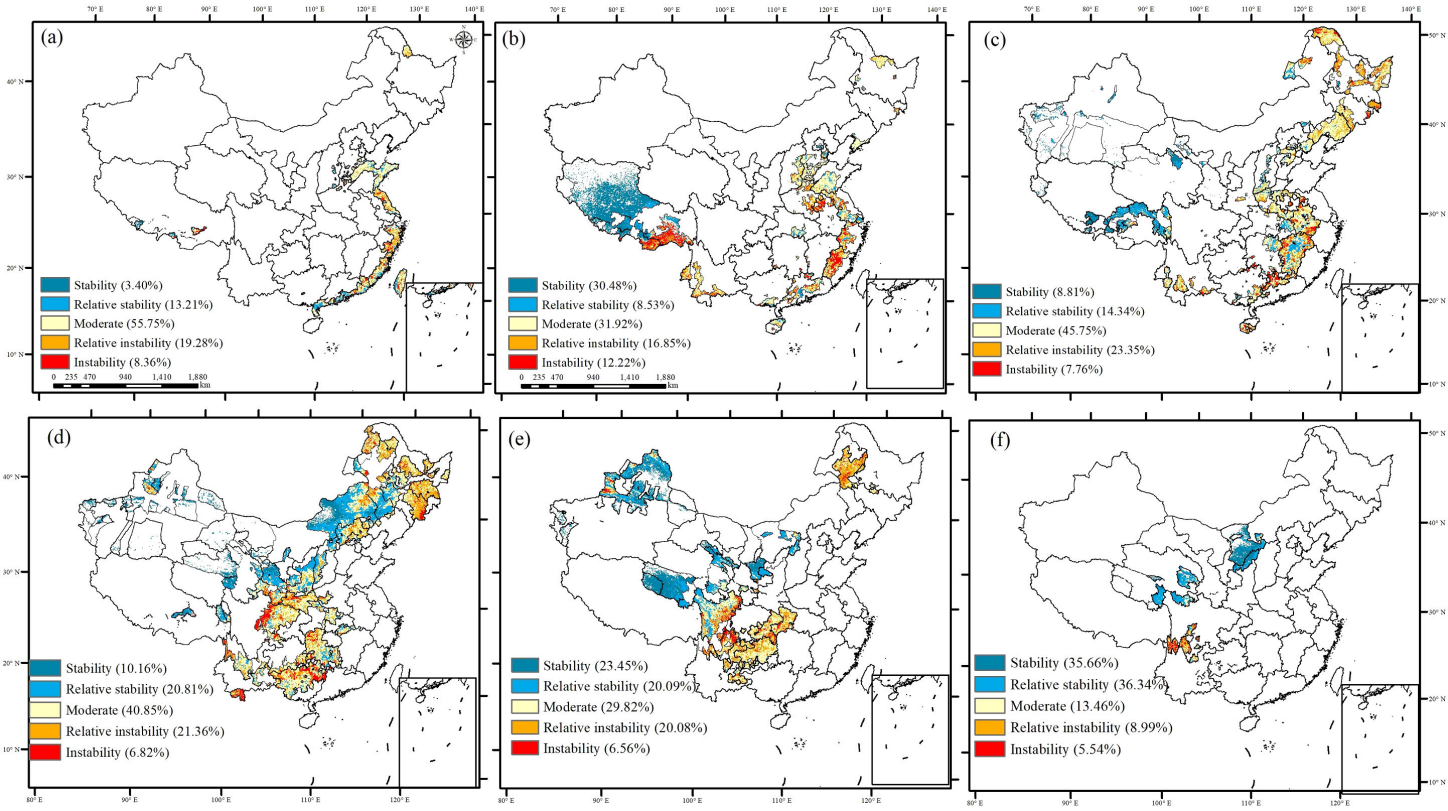
The stability of NEP from 1981 to 2019 in ecological project areas [**(A)** 0 ecological project areas; **(B)** 1 ecological project area; **(C)** 2 ecological project areas; **(D)** 3 ecological project areas; **(E)** 4 ecological project areas; **(F)** 5 ecological project areas].

In the ecological engineering areas, the highest percentage of stable carbon sinks were located in regions where five overlapping ecological engineering initiatives were implemented, and the stability of carbon sinks reached 35.66% (**Figure 8**). These areas were primarily located within the overlapping engineering regions, such as the Grain to Green Project, Natural Forest Resource Protection Project, Three-River Ecological Conservation and Restoration Project, and Three-North Shelter Forestation Project areas. The second-highest percentage of stable carbon sinks were primarily concentrated in regions with a single ecological implementation area, namely, the Return Grazing Lands to Grasslands Project area in Tibet. The regions with a high percentage of unstable carbon sinks were mainly located in areas with the overlapping implementation of two or three ecological projects, such as the northeastern and southeastern parts of China. This suggests that the implementation of five overlapping ecological projects contributed to the increase in the stability of carbon sinks.

### 3.3 Relative importance of driving factors of the stability of carbon sinks

**Figure 9:**
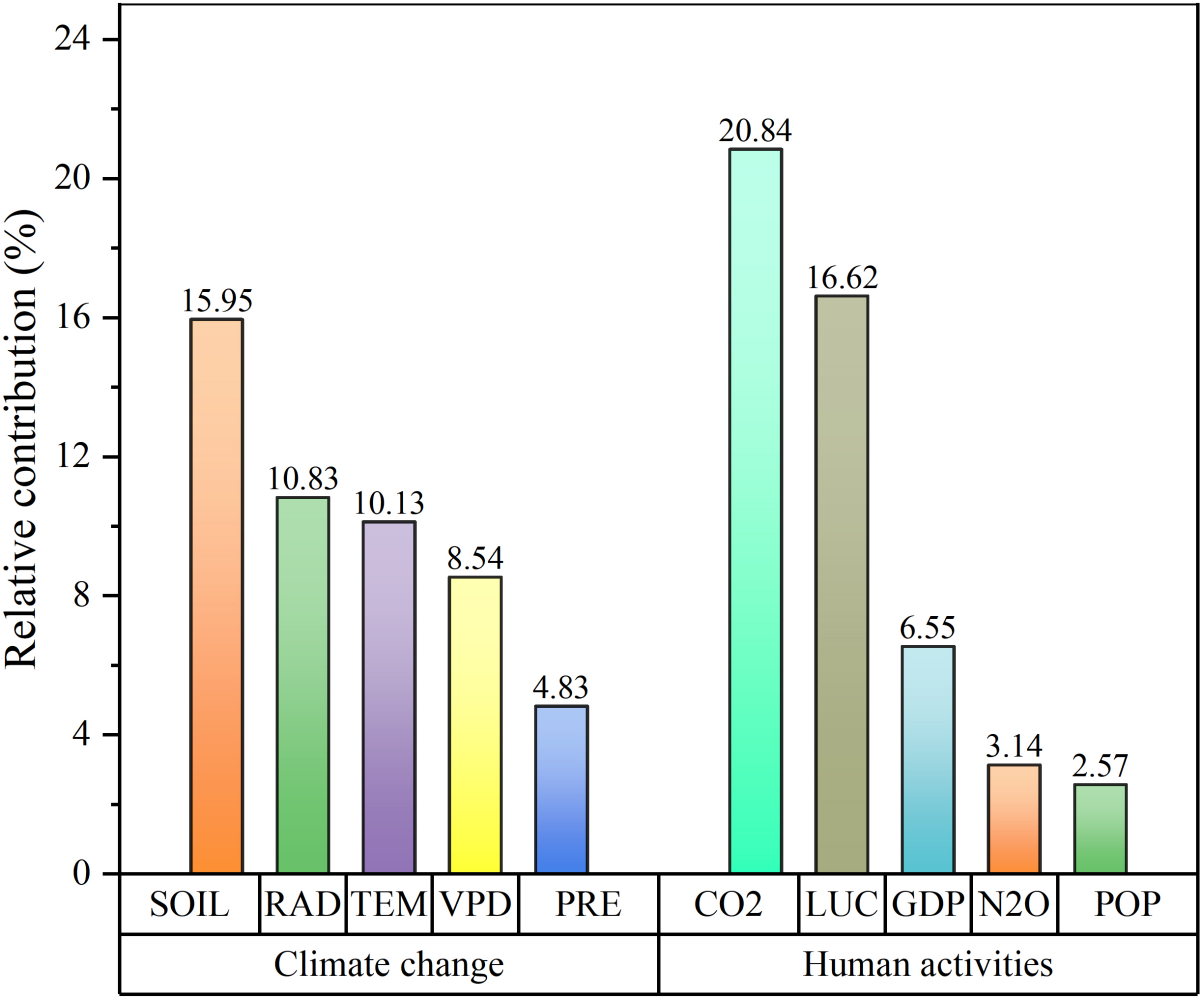
The relative importance of driving factors on the carbon sinks in China (VPD, vapor pressure deficit; TEM, air temperature; PRE, precipitation; SOIL, soil moisture; LUC, land use change; POP, population density; GDP, gross domestic product).

Based on the BRT model, climate change and human activities made comparable contributions to the stability of carbon sinks, accounting for 50.28% and 49.72%, respectively (**Figure 9**). The soil moisture, solar radiation, and air temperature were relatively important climatic factors, accounting for 15.95%, 10.83%, and 10.13%, respectively, whereas the VPD and precipitation had less significant effects. The anthropogenic CO_2_ emissions and LUC were significant anthropogenic factors, contributing 20.84% and 16.62%, respectively, whereas the GDP, anthropogenic N_2_O emissions, and population density (POP) were less important. This suggests that the soil moisture content and CO_2_ emissions primarily drove the increase in carbon sinks.

**Figure 10:**
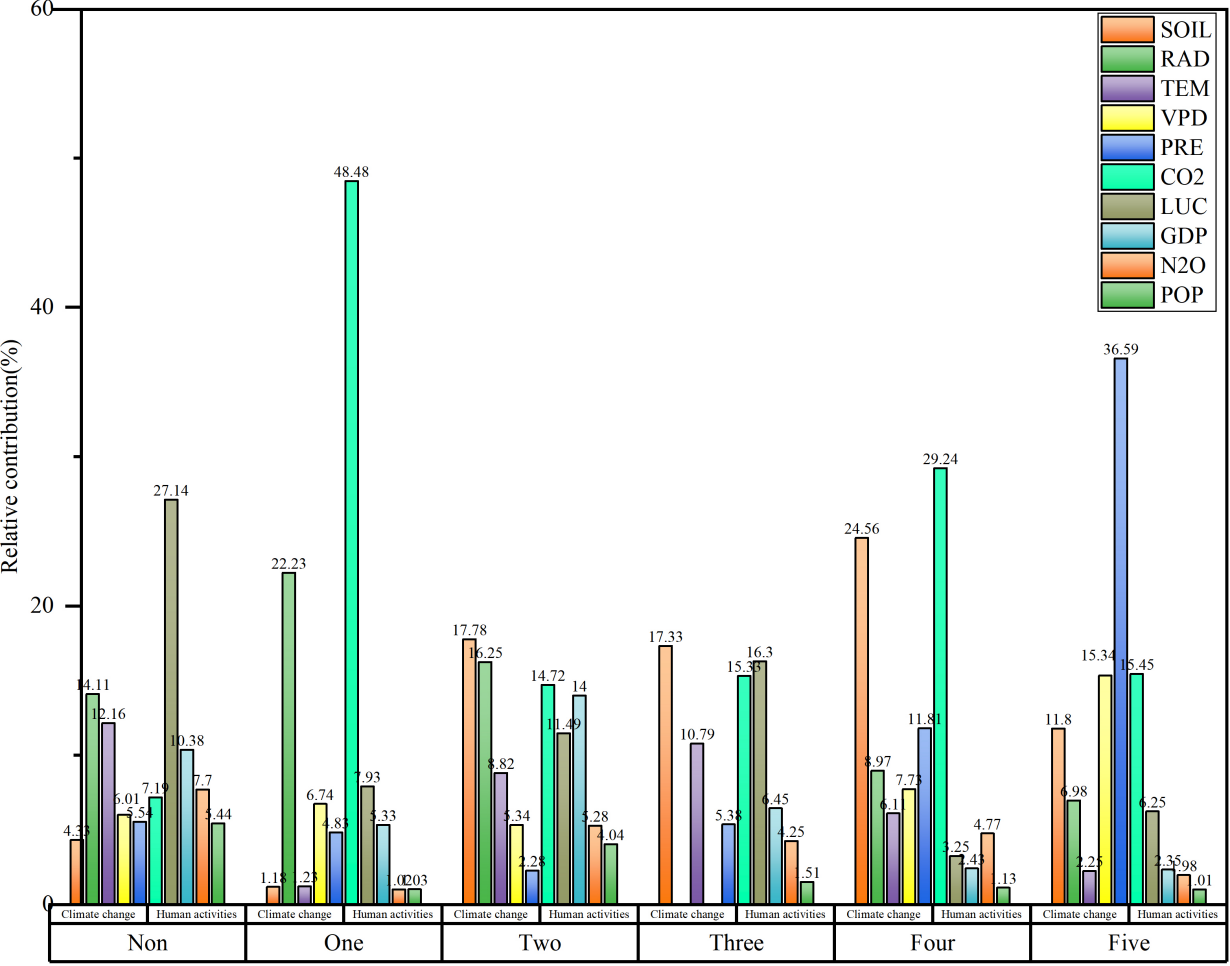
The relative importance of driving factors on the carbon sinks in ecological project areas (Orange color: human activity factors; Green color: climate change factors; Non: 0 ecological project areas; One: 1 ecological project area; Two: 2 ecological project areas; Three: 3 ecological project areas; Four: 4 ecological project areas; (VPD, vapor pressure deficit; TEM, air temperature; PRE, precipitation; SOIL, soil moisture; LUC, land use change; POP, population density; GDP, gross domestic product).

Human activities had a minimal impact on the carbon sinks in the eastern region where either no ecological engineering projects or only a single project was implemented (**Figure 10**). In the regions with one ecological engineering project, human activities contributed 63.90% to the importance of the carbon sinks, and the CO_2_ emissions were the primary contributor. With increasing number of overlapping ecological projects, the climate factors contributed more to the carbon sinks. In areas such as the Yellow River Basin and the Three Rivers region, where five ecological projects were implemented, climate change contributed 72.96% to the carbon sinks, and precipitation was the most significant factor, contributing 36.59%. Conversely, in the three or four ecological projects overlapped, the soil moisture content made a relatively high contribution, accounting for 17.33% and 24.56%, respectively.

### 3.4 Prediction of carbon sinks in overlapping ecological project areas

**Figure 11:**
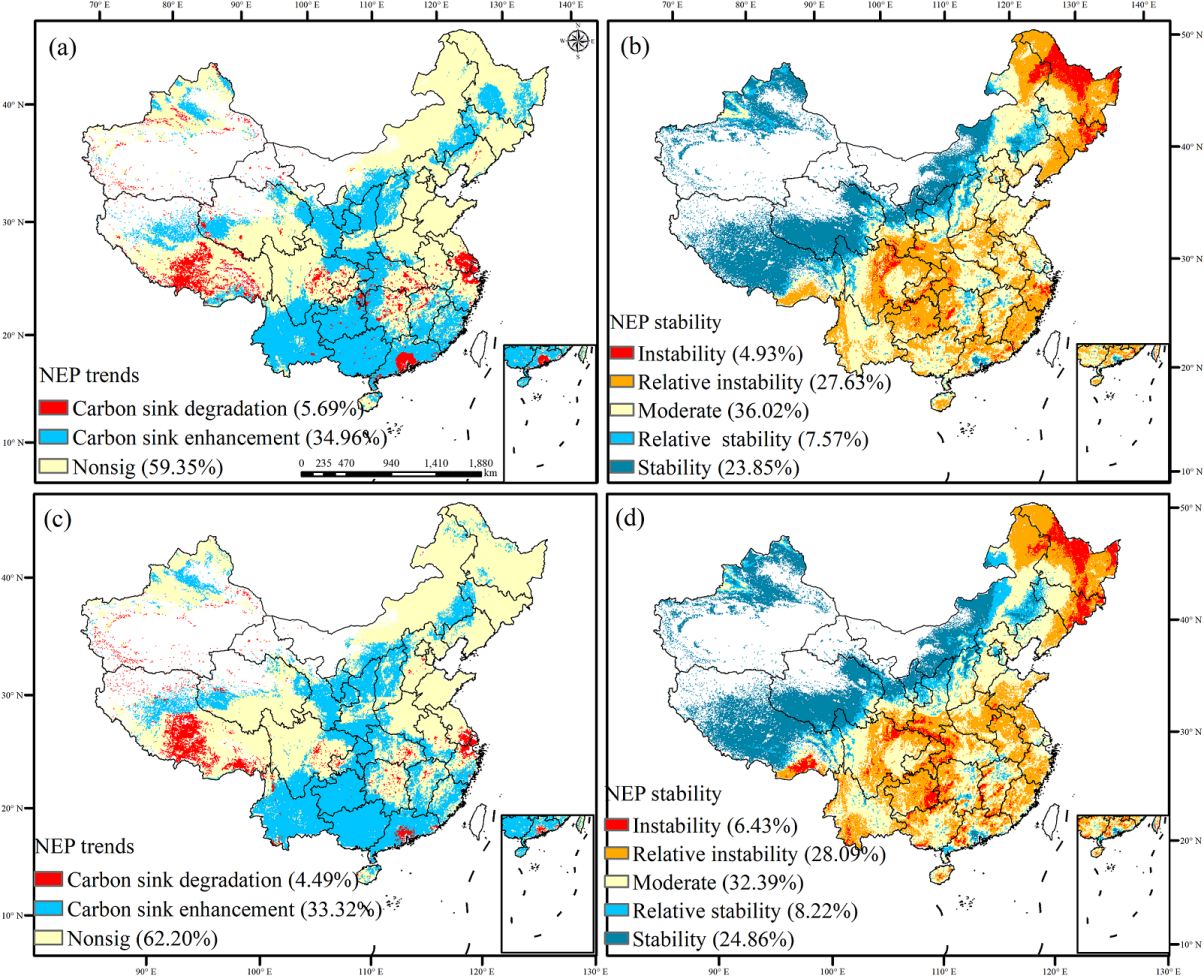
Trend and stability of carbon sink under the SSP126 scenario in 2060 and 2100 [**(A, B)** the trend and stability of carbon sink in 2060; **(C, D)** the trend and stability of carbon sink in 2100].

Under SSP126, the degradation of carbon sinks decreases from 5.69% to 4.49% between 2060 and 2100, and the most significant reductions occur in Tibet, the Yangtze River belt, and the Pearl River region (**Figure 11**). Conversely, the enhancement of carbon sinks undergoes relatively minor changes, accounting for 34.96% and 33.32% in 2060 and 2100, respectively, and these changes are primarily concentrated in the Yellow River belt and Southwest China. The stability of carbon sinks changes by 23.85% and 24.86% in 2060 and 2100, respectively, and these changes predominantly occur in northwest China and Tibet. Conversely, the instability of carbon sinks changes by 4.93% and 6.43% in 2060 and 2100, respectively, and these changes primarily occur across northeast and southwest China. Under SSP126, rapid technological advancements, the promotion of sustainable economic growth by economic globalization, the development of low-carbon energy technologies, alongside reduced energy intensity and increased environmental awareness, and the widespread adoption of renewable energy sources collectively contribute to reducing the degradation of carbon sinks. As a result, the carbon sinks shift toward greater stability.

**Figure 12:**
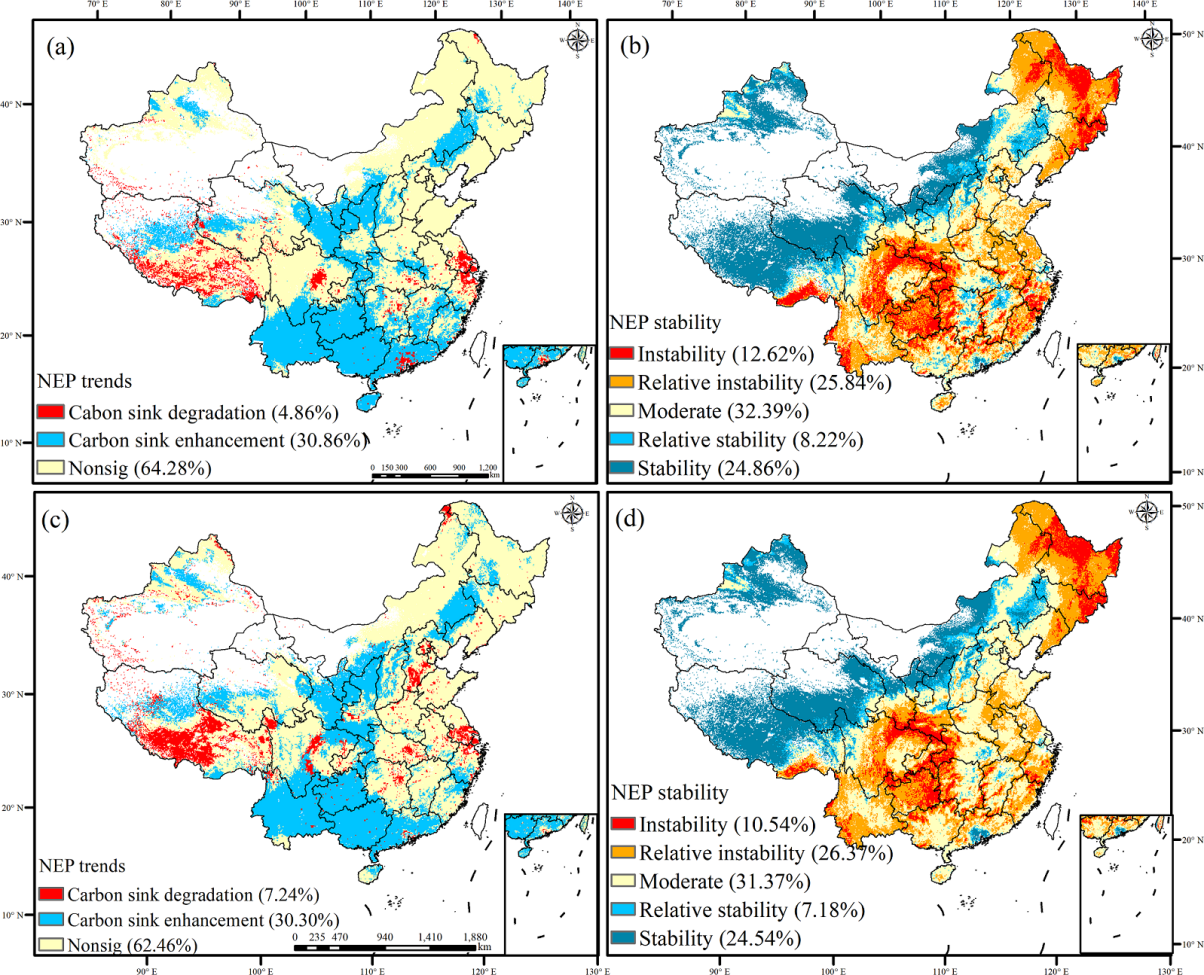
Trend and stability of carbon sink under the SSP585 scenario in 2060 and 2100 [**(A, B)** the trend and stability of carbon sink in 2060; **(C, D)** the trend and stability of carbon sink in 2100].

Under SSP585, the degradation of carbon sinks increases from 4.86% to 7.24% between 2060 and 2100 and is primarily driven by significant shifts in previously stable regions (**Figure 12**). The most notable changes occur in Tibet and the Beijing-Tianjin-Hebei area. The carbon sinks exhibit a significantly higher rate of decline by 2100 under SSP585 than the trends of carbon sinks from 1982 to 2019, particularly in the Tibetan Plateau region where the carbon sinks experience considerable degradation. Conversely, the percentage of carbon sink change remains relatively stable, and these changes primarily occur in the Loess Plateau and southwestern China. The stability of carbon sinks between 2060 and 2100 exhibits minimal variations, and the stable carbon sinks in northwest China experience insignificant changes. The regions with unstable carbon sinks are primarily located in southwest China, which are characterized by an increasing trend of carbon sinks but face heightened degradation risks. SSP585 includes a high reliance on conventional energy sources. As a result, the carbon sinks persist in a degraded state and exhibit increased instability.

**Figure 13:**
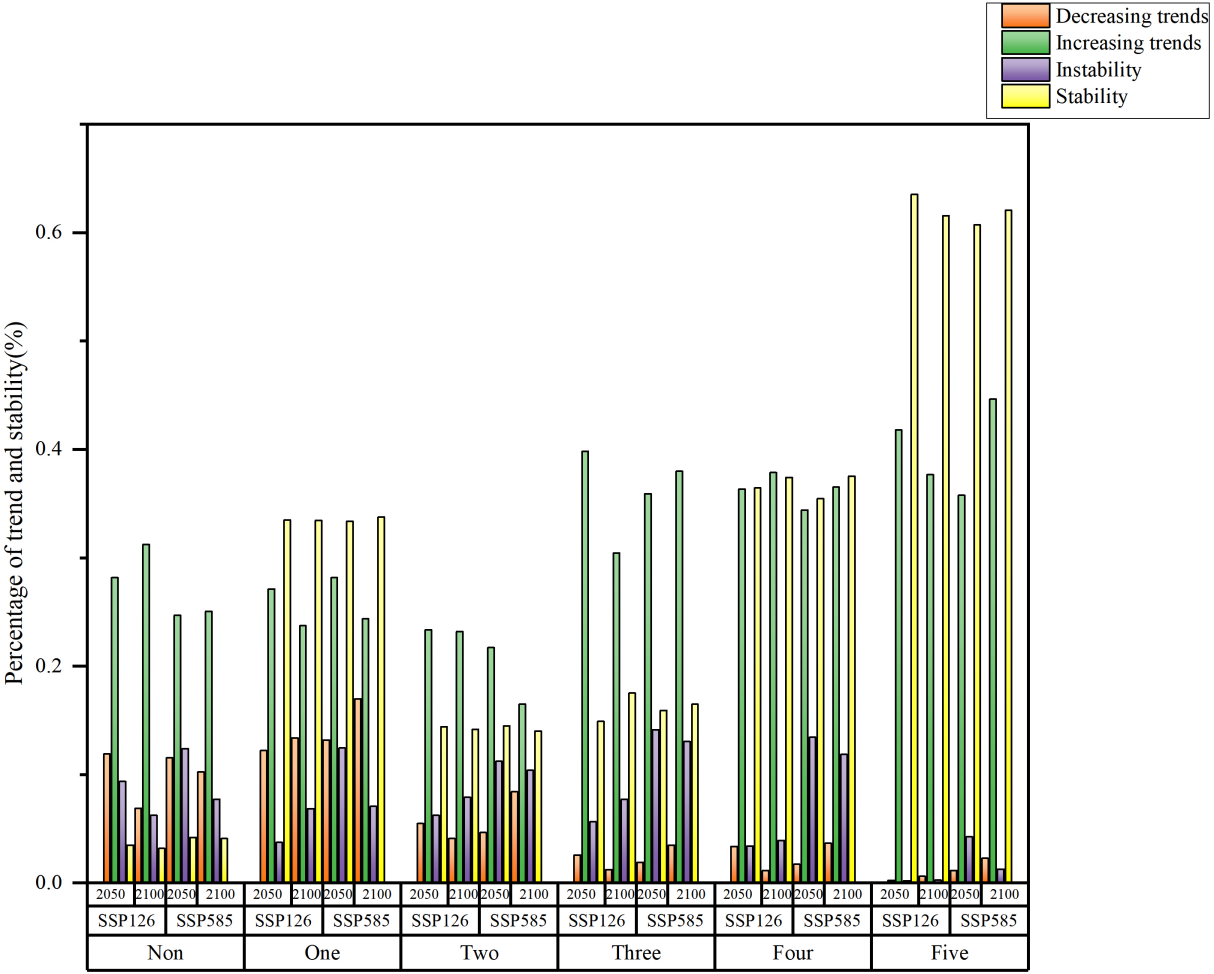
Trend and stability of carbon sink under the SSP126 and SSP245 scenarios in 2060 and 2100 in different ecological projects.

The areas with no, one, or two ecological engineering projects exhibit a higher proportion of carbon sink degradation (**Figure 13**). Conversely, in the regions influenced by four or five overlapping ecological engineering projects, the proportion of carbon sink enhancement increases with increasing number of projects. Under SSP126, by 2100, the Loess Plateau and Three-River region, affected by five overlapping ecological engineering projects, exhibit a carbon sink enhancement proportion of 41.80%, and the proportion of degraded carbon sinks is only 0.61%. The proportion of carbon sink stability increases with increasing number of overlapping ecological projects, aligning closely with the observed trend. By 2060, under SSP126, the areas with five overlapping ecological projects exhibit a stability proportion of 62.06%, and the instability is only 0.25%. The proportion of areas with decreasing trends of carbon sinks is higher under SSP585 than under SSP126.

## 4 Discussion

### 4.1 Trend and stability of carbon sinks in overlapping ecological engineering projects

Previous studies have used various methodologies, such as Empirical Mode Decomposition (EMD), Ensemble Empirical Mode Decomposition (EEMD), and wavelet analysis, to investigate the intricate dynamics of carbon sinks (Seddon et al., 2016; Liu et al., 2023c). Nevertheless, the complex interplay between climate change and anthropogenic activities results in significant interannual variability and instability of carbon sinks (Wang et al., 2023). This variability often manifests as spatial differences between the observed trends and the underlying stability of carbon sinks (Liu et al., 2023b). In addition, it is not clear whether overlapping ecological projects can contribute more to the increase in carbon sinks than single ecological projects (Cai et al., 2020). In this study, we addressed this research gap by conducting an in-depth examination of carbon sink stability. Our findings revealed that the regions in the east, characterized by either the absence of ecological projects or minimal overlap of projects tended to exhibit relatively weaker carbon sink stability. In contrast, areas such as the Loess Plateau and the Three-River source region, characterized by multiple overlapping ecological projects, had enhanced carbon sink stability. This observed variability underscores the critical importance of strengthening ecosystem carbon sink functions and customizing carbon neutrality strategies to suit specific regional conditions (Wang et al., 2022b). In areas with stable carbon sinks, maintaining existing ecological measures is essential for increasing the capacity and stability of carbon sinks. Conversely, in areas with less stable terrestrial carbon sinks, measures that must be taken include reducing anthropogenic disturbances, expanding ecological measures, and implementing diversified restoration strategies, all of which are aimed at enhancing the stability of carbon sinks and ensuring their long-term sustainability (Wang et al., 2023).

### 4.2 Driving mechanism of carbon sinks

Enhancing carbon sinks in terrestrial ecosystems requires a thorough understanding of the responses of carbon sink stability to climatic variations and human activities (Wang et al., 2022a, 2022). On the national scale, the impacts of human activities and climate change on the stability of carbon sinks are almost equal. Among the various anthropogenic factors, the CO_2_ emissions and LUC are especially critical in determining carbon sink stability. Regarding the climate factors, the soil moisture content and solar radiation are key determinants of carbon sink stability. Human activities have a substantial impact in regions such as the North China Plain, the Northeast China Plain, and the eastern coastal areas where the stability of carbon sinks is relatively low. The impacts of LUCs and anthropogenic CO_2_ emissions are particularly noteworthy in areas along the eastern coast and on the North China Plain and Northeast China Plain where ecological interventions are minimal (Zheng et al., 2019). The eastern region, characterized by intensive human disturbances, has undergone rapid economic development, which has been frequently accompanied by increased energy consumption and accelerated industrialization, resulting in substantial carbon emissions (Wang et al., 2022a). Industrial processes, transportation, and energy production significantly contribute to CO_2_ emissions, a potent greenhouse gas (Wu et al., 2021). Concurrently, rapid urbanization and land development activities lead to widespread deforestation, wetland degradation, and conversion of land to urban or agricultural land, reducing carbon stocks in ecosystems and diminishing the overall carbon sink capacity (Zhu et al., 2021).

The greater the number of overlapping ecological projects is, the more significant contribution of climate change to the stability of carbon sinks is, particularly in areas where five ecological projects overlap, in which the relative importance of the climate reaches 72.96%. In regions where five ecological engineering projects overlap, the ecological environments frequently exhibit increased fragility and greater susceptibility to the impacts of climate change. This increased sensitivity implies that variations in climatic conditions may result in more substantial and widespread consequences for the ecosystem, including alterations of the vegetation pattern, loss of biodiversity, and modification of the hydrological cycle (Fan et al., 2015). Given the inherent fragility of these ecological environments, the implementation of ecological engineering initiatives in such areas requires heightened consideration of climatic factors. Two primary rationales underscore the increased impact of climate change on carbon sequestration: 1) Climate fluctuations have the potential to cause substantial transformation within ecosystems, thereby affecting the stability and efficiency of carbon sequestration. For example, rising temperatures may trigger vegetation changes and may prolong the growing season, thereby affecting plant growth and the carbon absorption capacity (Wang et al., 2022a). 2) Regions characterized by ecological vulnerability are generally more susceptible to the adverse effects of extreme climate events, such as droughts and floods, which can result in abrupt and severe repercussions for carbon sequestration (Wang et al., 2021). Consequently, when implementing ecological engineering initiatives in these areas, particular emphasis must be placed on climatic factors, and measures should be adopted to mitigate the impacts of climate change on carbon sequestration (Ma et al., 2019).

In the northwest and southwest regions of China, where three or four ecological projects overlap, the contribution of soil moisture to carbon sink stability is relatively high, accounting for 17.33% and 24.56%, respectively. In the southwestern karst region, characterized by uneven distributions of water and soil resources and hydrological fluctuations, the rate of soil formation is slow, the water retention capacity is low, and the potential for ecological restoration is low (Wang et al., 2019). Previous studies have found that insufficient soil moisture and a high atmospheric VPD are the primary factors hindering vegetation greening in karst areas, which is consistent with the result with our study (Yue et al., 2022). Drought-induced stress resulting from water scarcity has the potential to impede the recovery and sustained growth of karst ecosystems, making ground vegetation productivity highly sensitive to climate change-driven variations in vapor pressure deficit (Qiao and Xia, 2024). Furthermore, human activities, such as terracing and deforestation, exacerbate the vulnerability of already fragile karst ecosystems, particularly in areas with slopes of greater than 25° (Chen et al., 2023). Ensuring adequate soil moisture, which is crucial for supporting vegetation growth, is imperative, particularly in semi-arid regions. Global climate change is exacerbating the decline in soil moisture across numerous regions, primarily due to reduced precipitation and increased evaporation of soil caused by increased temperatures (Yue et al., 2020).

The northwest region of China, where one or two ecological projects overlap, plays a significant role in global climate change and is characterized by complex ecological systems. Previous research has highlighted the significant relationship between carbon sinks and soil moisture dynamics and has identified the decline in soil moisture as a primary driver of carbon sink reduction (Song et al., 2022; Du et al., 2024). Soil moisture plays a crucial role in supporting optimal vegetation growth in semi-arid and arid environments (Zhou et al., 2018). Consequently, insufficient soil moisture restricts sustained plant growth in the arid regions of northwest China, increasing the vulnerability of carbon sinks to climatic variations (Qiao and Xia, 2024). The prevailing climate in this region is predominantly a temperate continental climate, and moisture availability significantly influences vegetation dynamics in this region. Decreasing precipitation levels contribute to increases in temperatures and radiation exposure, thereby intensifying drought conditions (Piao et al., 2020).

Therefore, in response to factors affecting the stability of carbon sinks, appropriate measures should be implemented (Chen and Yu, 2019). In ecological engineering zones that are highly sensitive to climate dynamics, enhancing meteorological disaster warning systems, establishing emergency response mechanisms, and implementing disaster prevention measures effectively are imperative for strengthening the resilience of ecosystems and societies against catastrophic events (Piao et al., 2022b). For instance, it is essential to improve the management of water conservancy irrigation and drainage projects in conjunction with advanced weather forecasting to mitigate the effects of sudden precipitation fluctuations on soil moisture levels and reduce the occurrence of extreme climate events (Chen and Yu, 2019). In regions heavily affected by human activities, comprehensive soil and water conservation initiatives and ecological restoration projects should be implemented, with strict adherence to ecological protection boundaries and the preservation of biodiversity (Wang et al., 2024). Furthermore, strengthening the establishment of nature reserves is crucial for mitigating forest mortality caused by extreme climate events, thereby improving carbon sink stability.

### 4.3 Sustainability of carbon sinks in overlapping ecological engineering project areas

Carbon sink forecasting is crucial for the development of effective climate policies by governments and international organizations (Xu et al., 2022a). By understanding the trends and stability of carbon sinks, targeted emission reduction measures and carbon trading policies can be developed to more effectively address climate change (Zhang and Hanaoka, 2021). Current research has predominantly focused on short-term fluctuations in carbon sequestration rates, and long-term forecasts that consider various climate and land use scenarios are lacking (Xu et al., 2022a). Long-term predictions can quantify interannual variations and reveal trends, thereby providing a theoretical basis for the development of systematic carbon reduction strategies and providing technical support for climate change mitigation efforts (Piao et al., 2020; Wu et al., 2024). In this study, we projected future trends of carbon sinks in China under SSP126 and SSP585 scenarios. our research results indicated that by 2100, under SSP126 scenario, carbon sinks in southern China and the Yellow River Basin will expect to exhibit a stable increasing trend. This suggests that ecosystems in these regions will gradually recover, with continuous improvement in the carbon absorption capacity. Conversely, the carbon sinks in Tibet are projected to exhibit a stable degradation, indicating persistent deterioration of ecosystem health and a gradual decrease in the carbon capacity of carbon. Additionally, the Yangtze River Basin is expected to experience an unstable decreasing carbon sink trend, likely due to environmental changes and human activities that cause fluctuations in the carbon absorption capacity (Xu et al., 2023b).

Under SSP585 scenario, the proportions of degraded and unstable carbon sinks will significantly increase, particularly in Tibet and economically developed urban areas such as the Yangtze River Delta and the Beijing-Tianjin-Hebei region. The carbon sinks in these areas will not only severely degraded but will also become extremely unstable, indicating that, under this high-emission scenario, ecosystems face greater stress and challenges (Nooni et al., 2021). As major economic centers of China, the Yangtze River Delta and the Beijing-Tianjin-Hebei region have dense populations and frequent industrial activities, which lead to high carbon emissions. Under this high-emission scenario, these factors will further exacerbate the degradation and instability of carbon sinks. In regions characterized by the presence of only one or two overlapping ecological projects, such as southwestern and northeastern China, the stability of carbon sink will exhibit conspicuous inadequacy. This deficiency primarily arises from the slow economic development and a pronounced reliance on conventional energy sources. These factors impede efforts to stabilize carbon sinks, particularly within the constraints of limited ecological interventions (Yue et al., 2022). In contrast, locations such as the Loess Plateau and the Three-River region, where multiple ecological initiatives are concurrently being implemented, will derive substantial advantages from the synergistic interplay among these initiatives, thus significantly bolstering both the stability and efficacy of carbon sinks (Niu et al., 2022). Consequently, these regions manifest elevated proportions of both stable and enhanced carbon sinks under SSP126 and SSP585.

Therefore, the implementation of significant ecological engineering projects should thoroughly account for the limiting factors imposed by climatic conditions, thereby avoiding the applications of singular ecological projects or isolated restoration measures (Niu et al., 2022). Moreover, it is crucial to fully harness the synergistic potential inherent in a comprehensive array of ecological engineering interventions (Shao et al., 2023). This approach strengthens the ecosystem’s capacity to withstand endure external disruptions, including natural disasters and climate fluctuations, thereby enhancing the ecosystem’ resilience and stability (Wang et al., 2024). Accelerating the trajectory of ecosystem recovery and expediting the attainment of ecological equilibrium can be facilitated through complementary actions.

## 5 Conclusions

Using the ensemble empirical mode decomposition method and machine learning algorithms (enhanced boosted regression trees), the aims of this study to elucidate the stability of carbon sinks and their driving mechanisms in areas where ecological projects overlap and to predict the potential enhancement in carbon sinks under varying climate and human activity scenarios. The findings revealed that: (1) The carbon sinks clearly and steadily increased in regions where five ecological projects were implemented from 1982 to 2019. In contrast, the carbon sinks did not significantly increase in regions with two or three ecological projects. (2) As the number of ecological projects increased, the impact of human activities on the carbon sinks gradually decreased. In eastern China, rapid economic development and significant interference from human activities hindered the growth of carbon sinks. In contrast, in western China, the warming and humidification trend of the climate, large-scale afforestation, and other ecological projects have significantly improved carbon sinks. (3) The regions with five overlapping ecological projects exhibited the greatest enhancement and stability of carbon sinks under different scenarios. Compared with the SSP585 scenario, under the SSP126 scenario, the carbon sinks increased, and their stability was greater.

## Data Availability

The original contributions presented in the study are included in the article/**Supplementary Material**. Further inquiries can be directed to the corresponding authors.
