# Supplementary material for: Assessment of the potential for carbon sink enhancement in the overlapping ecological project areas of China
Source: Front Plant Sci. 2024 Nov 26;15:1482077. doi: 10.3389/fpls.2024.1482077 (PMC11628300; doi:10.3389/fpls.2024.1482077)
Supplement: Supplementary file 1 [file Table1.docx]

Table S1 Model evaluation metrics (AUC) for the BRT model

| Run 1 | Run 2 | Run 3 | Run 4 | Run 5 | Run 6 | Run 7 | Run 8 | Run 9 | Run 10 | Average |
| --- | --- | --- | --- | --- | --- | --- | --- | --- | --- | --- |
| 0.91 | 0.89 | 0.87 | 0.90 | 0.85 | 0.90 | 0.88 | 0.87 | 0.86 | 0.90 | 0.88 |
